# Class 1 Integrons in Environments with Different Degrees of Urbanization

**DOI:** 10.1371/journal.pone.0039223

**Published:** 2012-06-22

**Authors:** Maximiliano Nardelli, Paula Marina Scalzo, María Soledad Ramírez, María Paula Quiroga, Marcelo Hernán Cassini, Daniela Centrón

**Affiliations:** 1 Laboratorio de Investigaciones de los Mecanismos de Resistencia a Antibióticos, Facultad de Medicina, Instituto de Microbiología y Parasitología Médica (IMPaM, UBA-CONICET), Universidad de Buenos Aires, Ciudad Autónoma de Buenos Aires, Argentina; 2 Grupo GEMA, Departamento de Ciencias Básicas, Universidad Nacional de Luján, Luján, Buenos Aires, Argentina; 3 Laboratorio de Biología del Comportamiento, IBYME, Ciudad Autónoma de Buenos Aires, Argentina; Universidad Nacional de La Plata., United States of America

## Abstract

**Background:**

Class 1 integrons are one of the most successful elements in the acquisition, expression and spread of antimicrobial resistance genes (ARG) among clinical isolates. Little is known about the gene flow of the components of the genetic platforms of class 1 integrons within and between bacterial communities. Thus it is important to better understand the interactions among “environmental” *intI1*, its genetic platforms and its distribution with human activities.

**Methodology/Principal Findings:**

An evaluation of two types of genetic determinants, ARG (*sul1* and *qacE1*/*qacE*Δ*1* genes) and lateral genetic elements (LGE) (*intI1*, IS*CR1* and *tniC* genes) in a model of a culture-based method without antibiotic selection was conducted in a gradient of anthropogenic disturbances in a Patagonian island recognized as being one of the last regions containing wild areas. The *intI1*, IS*CR1* genes and *intI1* pseudogenes that were found widespread throughout natural communities were not associated with urbanization (p>0.05). Each ARG that is embedded in the most common genetic platform of clinical class 1 integrons, showed different ecological and molecular behaviours in environmental samples. While the *sul1* gene frequency was associated with urbanization, the *qacE1*/*qacE*Δ*1* gene showed an adaptive role to several habitats.

**Conclusions/Significance:**

The high frequency of *intI1* pseudogenes suggests that, although *intI1* has a deleterious impact within several genomes, it can easily be disseminated among natural bacterial communities. The widespread occurrence of IS*CR1* and *intI1* throughout Patagonian sites with different degree of urbanization, and within different taxa, could be one of the causes of the increasing frequency of multidrug-resistant isolates that have characterized Argentina for decades. The flow of ARG and LGE between natural and clinical communities cannot be explained with a single general process but is a direct consequence of the interaction of multiple factors operating at molecular, ecological, phylogenetic and historical levels.

## Introduction

In addition to the global causes of death by viruses, bacteria and parasites, which are a huge burden on public health, the progressive increase of multidrug resistance in all geographical regions has been identified as a public health priority according to the World Health Organization, 2011 (http://www.who.int/drugresistance). In recent years, research on the function of antibiotic resistance in non-clinical environments has begun to receive attention [1], [2], [3], [4]. This interest is based on the idea that a better understanding of the diversity of patterns and biological functions of antibiotic resistance mechanisms may eventually help to control its threats towards human and also animal health. The role of the environment as a reservoir of strains that have never before been isolated from humans was demonstrated during the outbreak caused by enteroaggregative *Escherichia coli* that had acquired the genes to produce Shiga toxins in Germany in May 2011. This episode also stresses the negative consequence of having mechanisms of antimicrobial resistance in these isolates (*bla*_TEM-1_ and *bla*_CTX-M-15_), which most likely helped the bacteria to survive and persist in different habitats [5] **.**

Most of the new research into natural bacterial communities has focused on antimicrobial resistance genes (ARG) that confer resistance to antimicrobial drugs, mainly associated with protection against natural antibiotics or with functional properties among the metabolic pathways of environmental bacteria [2], [6], [7]. In contrast, scarce research has focused on the natural occurrence and role of the genetic platforms (transposons, integrons) that participate in the capture and dispersion of these genes within and among genomes, usually known as mechanisms of lateral genetic transfer [8]. To our knowledge, there are no prospective studies that analyses the independent occurrence of genetic components of a particular genetic platform, i.e. the lateral genetic transfer determinants such as integrons or transposons with the ARG elements, considering both ecological and molecular parameters.

**Figure 1:**
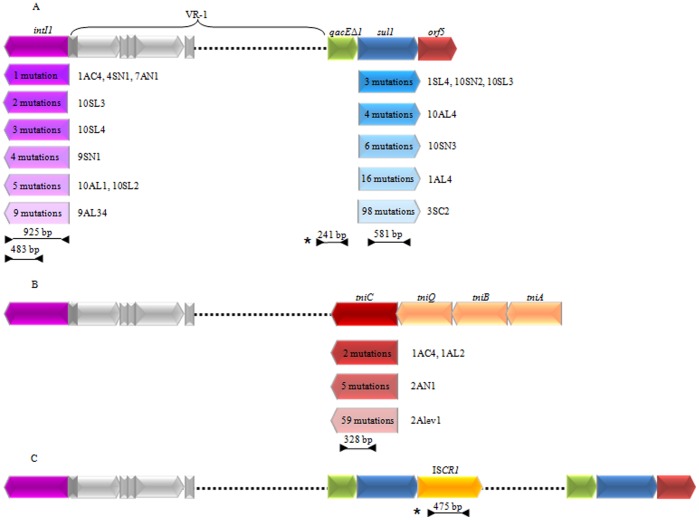
Genetic platforms class 1 integrons described in the clinical samples from Argentina. (A) The typical class 1 integron with the 3′-CS end containing *qacE*Δ*1*, *sul1* and *orf5*, (B) the unusual or complex class 1 integrons and, (C) the Tn*402*-type integrons. Arrows represent the different ORFs: the violet arrow exemplifies the *intI1* gene, the blue arrow represents the *sul1* gene, the green arrow stands for the *qacE*Δ*1* gene, the red arrow represents the *tniC* gene, and the yellow arrow represents the IS*CR1* gene. The degree of colour intensity indicates different alleles for the corresponding gene. Grey rectangles stand for *attCs* (light grey) and *attIs* (dark grey). The dotted lines show the variable region of class 1 integrons (VR-1 is variable region 1). Full lines between the arrowheads above the integron structure show the expected amplification products using the different primer combinations. In order to define the different alleles for the *intI1* genes the sequences were compared to AN AM412236 (isolates 1AC4, 4SN1, 9SN1 and 9AL34) and AN DQ247972 (isolates 7AN1, 11601AL, 11602SL, 11603SL and 11604SL). The *sul1* and *tniC* alleles show the percentage of identity to AN JF262166 and GQ857074, respectively. The *qacE1/qac*Δ*E1* and the IS*CR1* sequences (indicated with an asterisk) were 100% identical to the clinical alleles AN HM999792 and EU722351, respectively. The graphic is not drawn to scale.

Lateral genetic transference is a widespread phenomenon that is not only largely responsible for the ability of pathogenic and opportunistic bacteria to resist clinical antibiotic pressures [9], but also enables exchange of the accessory genome, which is a major contributor to bacterial evolution [10]. Of the different mechanisms involved in lateral genetic transfer, the class 1 integrons are one of the most successful elements in the acquisition, abundance, maintenance and spread of antimicrobial resistance gene cassettes among gram-negative bacilli isolated from clinical samples [11], [12], [13], [14]. Although their role has not been yet investigated, class 1 integrons have been also found in gram-positive clinical strains, including methicillin-resistant *Staphylococcus aureus* and *Corynebacterium* species [15], [16], [17], [18], [19], [20], from several hospitals around the world. The basic structure of an integron possesses a gene for an integrase (*intI*), a recombination site (*attI*) and a promoter (P_c_) that permits the expression of gene cassettes incorporated in the variable region [21]. Several genetic structures have been described at the 3′ end of the variable region of class 1 integrons [22], [23], [24], [25], [26], [27], [28]. There are three genetic platforms containing and spreading the class 1 integrons described in clinical samples from Argentina (Figure 1): (i) the most common one exhibits the well-known 3′-conserved segment (3′-CS) at the end of the variable region, which contains the *qacE*Δ*1* gene that is a deleted form of the quaternary ammonium compounds resistance gene cassette, *qacE*, followed by the *sul1* gene that confers resistance to sulphonamides, and finally the *orf5* of unknown function [12] (Figure 1A); (ii) the complete or incomplete module of Tn*402*, *tniC*-*tniQ*-*tniB*-*tniA* [29] (Figure 1B); and (iii) the 3′-CS can be invaded by the putative site-specific recombinase IS*CR1*, which adds a second variable region that can contain an important variety of antimicrobial resistance genes such as *bla*_CTX-M-2_ and *qnrB10*, which are known as unusual or complex class 1 integrons [30], [31], [32], [33] (Figure 1C). Neither class 1 integrons nor unusual class 1 integrons allow intracellular mobilization of the *intI1* gene *per se*. However, almost all clinical members of class 1 integrons harbour the IR of the Tn*402* transposon, transforming this genetic platform into a mobile element when the *tns* genes are provided *in trans* [25], [34]. In addition, Tn*402* targets plasmid and transposon resolution sites (*res*) [35], expanding the range of lateral gene transfer between clinical and natural communities [1]. It is very important from a clinical standpoint that the association of genes in the same genetic platform is co-selected under antibiotic pressure.

The relevant role of natural communities as a reservoir and original source of class 1 integrons was recently identified [1], [15], [36], [37]. Since then, their distribution has been reported in environments with different degrees of human disturbance [1], [15], [36], [37], [38], [39], [40], [41], [42]. Overall, it is assumed that 2.65% of eubacterial cells in non-clinical samples contain a class 1 integron [41]. However, factors involved in the distribution of non-clinical class 1 integrons within natural communities remain largely unknown. What is known with certainty is that the class 1 integrons confer a benefit to the host cell due to their ability to acquire gene cassettes that could provide advantages for survival in hostile environments [43], [44], [45], [46], [47].

Concerning the molecular evolution of these elements, class 1 integrons were found to be chromosomally located, pre-dating the association with the Tn*402*-like transposon in non-clinical samples, suggesting that the ancestor of the clinical class 1 integron was more like a typical chromosomal integron [1]. The understanding of the molecular and environmental properties that contribute to the global success of class 1 integrons is the first step towards compiling a comprehensive story of how genes, genetic platforms, bacterial populations and selection pressures interact with human activities. However, it is difficult to assess the directionality of the flow of genes among natural environment and human habitats. The different alleles of the *intI1* gene from natural communities led to the identification of the sources of both “environmental” and “clinical” class 1 integrons [48].

**Figure 2:**
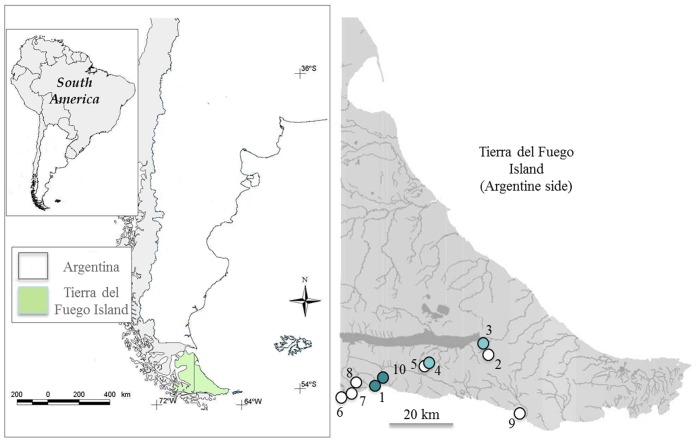
Study area. The geographic sites where the sampling was performed are numbered from 1 to 10 (see Table 1). The circles represent low (white), medium (light blue) and high (dark blue) degrees of urbanization.

The aim of this study was to analyse the relationships among “environmental” *intI1*, its genetic platforms and its distribution with human activities in areas with different levels of urbanization (Table 1). Our methodology was based on two strategies: (i) we worked at two scales of analyses, molecular and ecological levels, and (ii) we evaluated two types of genetic elements from the same samples, ARG (*sul1* and *qacE1* genes) and genetic elements associated to lateral genetic transfer such as *intI1*, IS*CR1* and *tniC* genes, called lateral genetic elements (LGE) for convenience in this paper and which comprise the genetic platforms of class 1 integrons. The first strategy allows the simultaneous analyses of ecological patterns and molecular mechanisms. The second strategy is based on the expectation that ARG will respond differently to LGE regarding the geographical variation of urbanization due to the different roles of these elements. We hypothesized that, if LGE serve as a general response mechanism to environmental stress, they should be present in both “clean” and urbanized habitats, as far as bacteria meet stressful conditions in “clean” habitats (for example, extreme seasonal or daily variations in weather conditions). Thus, they should present a weak relationship with the degree of urbanization. In contrast, ARG should be closely related to antibiotic pressure, and thus will present a strong link to geographical variations in urbanization and human presence. The field work was conducted in Tierra del Fuego, a Patagonian island from Argentina and Chile (Figure 2), which is recognized as being one of the last places on Earth that contains land areas that can still be considered wild or “clean”, given its great extensions of intact, natural vegetation and large vertebrate assemblages, along with a low human population density [49].

**Table 1:** Sampling sites of Tierra del Fuego Island.

Site	Name	Date	Geographic location	Houses	Roads	Other buildings	Urbanization
1	Ushuaia 1 (Pipo River)	22/01/06	S54° 49′ 58′′ W68° 21′ 05′′	>100	>10	10	High
2	Road N°26 1 (Turbio River)	25/01/06	S54° 47′ 37′′ W67° 15′ 36′′	0	1	0	Low
3	Road N°26 2 (Turbera Maucasen)	25/01/06	S54° 35′ 33′′ W67 03′ 38′′	6	1	2	Medium
4	Escondido Lake 1 (hotel)	27/01/06	S54° 40′ 49′′ W67° 48′ 48′′	15	2	2	Medium
5	Escondido Lake 2 (stream)	27/01/06	S54° 41′ 2′′ W67° 49′ 39′′	0	2	0	Low
6	National Park 1 (Ovando River)	01/02/06	S54° 50′ 36′′ W68° 34′ 42′′	0	2	0	Low
7	National Park 2 (Ensenada Bay stream)	01/02/06	S54° 50′ 48′′ W68° 28′ 56′′	0	1	1	Low
8	National Park 3 (Pipo River)	02/02/06	S54° 49′ 2′′ W68° 28′ 37′′	0	1	0	Low
9	Baliza Davidson stream (Moat)	04/02/06	S54° 56′ 24′′ W66° 54′ 30′′	0	1	0	Low
10	Ushuaia 2 (Ushuaia River)	20/01/06	S54° 47′ 37′′ W68° 15′ 36′′	>100	>10	10	High

Degree of urbanization was quantitatively estimated by counting the number of buildings and roads that were contained within a 1 km circular area around each site (see Material and Methods).

## Results and Discussion

### Taxonomic Distribution of the Components of the Genetic Platforms of Class 1 Integrons

We identified the following bacterial taxa using culture-dependent methods: γ and β classes within the Proteobacteria phylum (74 and 11 isolates, respectively), the Flavobacteria class within the Bacteroidetes phylum (3 isolates), *Arthrobacter, Streptomyces, Microbacterium* and *Micrococcus* genera within the Actinobacteria phylum (9 isolates) and the *Paenibacillus* genus within the Firmicutes phylum (1 isolate). Whole *intI1* genes were identified in 11 isolates of γ-proteobacteria (Table 2). Although *intI1* genes were more abundant in *Pseudomonaceae* than among other families of the γ-proteobacteria (6/11), *Enterobacteriaceae intI1* alleles showed high sequence diversity (Table 2). The *intI1* pseudogenes were mostly found in γ-proteobacteria, but they were also identified in two β-proteobacteria isolates and in one Actinobacteria isolate, evidencing the widespread dissemination of this genetic element. The remaining genetic determinants showed different patterns of taxonomic distribution as IS*CR1* and *sul1* were found in γ-proteobacteria and in Actinobacteria, and *tniC* in γ-proteobacteria and Flavobacteria (Table 2). The *qacE1/qacE*Δ*1* gene showed different frequencies between taxa (Table 3). Its frequency in γ-proteobacteria (18/74) was almost half of the frequency in the other taxa (10/23).

**Table 2:** Genetic features of samples isolated in this study.

Isolate	Genus	Taxonomic Class	*intI1*	Mutations^a^	*intI1* pseudogene	*qacE/qacE*Δ*1*	*sul1*	IS*CR1*	*tniC*
1AC1	*Arthrobacter*	Actinobacteria	0		0	0	0	0	0
1AC2	*Aeromonas*	γ- proteobacteria	**1**	**0**	0	0	0	0	0
1AC3	*Vibrio*	γ- proteobacteria	0		**1**	0	0	0	0
1AC4	*Vibrio*	γ- proteobacteria	**1**	**1**	0	**1**	0	0	**1**
1AC5	*Aeromonas*	γ- proteobacteria	0		0	0	**1**	**1**	0
1AL1	*Enterobacter*	γ- proteobacteria	0		0	0	**1**	0	0
1AL2	*Aeromonas*	γ- proteobacteria	0		0	**1**	0	0	**1**
1AL3	*Aeromonas*	γ- proteobacteria	0		0	0	0	0	0
1AL4	*Streptomyces*	Actinobacteria	0		0	**1**	**1**	0	0
1AL5	*Microbacterium*	Actinobacteria	0		0	**1**	0	**1**	0
1ALev1	*Arthrobacter*	Actinobacteria	0		0	0	0	0	0
1SC1	*Arthrobacter*	Actinobacteria	0		0	**1**	0	0	0
1SC2	*Arthrobacter*	Actinobacteria	0		0	**1**	0	0	0
1SC3	*Arthrobacter*	Actinobacteria	0		**1**	0	0	0	0
1SL1	*Pseudomonas*	γ- proteobacteria	0		**1**	0	0	**1**	0
1SL2	*Pseudomonas*	γ- proteobacteria	0		**1**	0	0	0	0
1SL3	*Arthrobacter*	Actinobacteria	0		0	**1**	0	0	0
1SL4	*Pseudomonas*	γ- proteobacteria	0		**1**	0	**1**	0	0
1SL5	*Pseudomonas*	γ- proteobacteria	**1**	**0**	0	0	0	0	0
1SLev1	*Micrococcus*	Actinobacteria	0		0	**1**	0	0	0
2AC1	*Janthinobacterium*	β- proteobacteria	0		**1**	0	0	0	0
2AC2	*Pseudomonas*	γ- proteobacteria	0		0	0	0	**1**	0
2AC3	*Pseudomonas*	γ- proteobacteria	0		**1**	0	0	**1**	0
2AL1	*Janthinobacterium*	β- proteobacteria	0		**1**	0	0	0	0
2AL2	*Janthinobacterium*	β- proteobacteria	0		0	0	0	0	0
2AL3	*Yersinia*	γ- proteobacteria	0		**1**	**1**	0	0	0
2AL4	*Pseudomonas*	γ- proteobacteria	0		**1**	0	**1**	0	0
2AL5	*Janthinobacterium*	β- proteobacteria	0		0	0	0	0	0
2ALev1	*Pseudomonas*	γ- proteobacteria	0		0	0	**1**	0	**1**
2AN1	*Flavobacterium*	Flavobacteria	0		0	0	0	0	**1**
2AN2	*Flavobacterium*	Flavobacteria	0		0	0	0	0	0
2AN3	*Paenibacillus*	Bacilli	0		0	0	0	0	0
3AL1	*Janthinobacterium*	β- proteobacteria	0		0	**1**	0	0	0
3AL2	*Serratia*	γ- proteobacteria	0		0	0	**1**	0	0
3AL3	*Yersinia*	γ- proteobacteria	0		0	0	0	0	0
3AL4	*Burkholderia*	β- proteobacteria	0		0	0	0	0	0
3ALev1	*Pseudomonas*	γ- proteobacteria	0		0	0	0	0	0
3ALev2	*Pseudomonas*	γ- proteobacteria	0		**1**	0	0	0	0
3ALev3	*Pseudomonas*	γ- proteobacteria	0		0	0	0	0	0
3AN1	*Pseudomonas*	γ- proteobacteria	0		0	0	0	0	0
3AN2	*Pseudomonas*	γ- proteobacteria	0		**1**	0	**1**	**1**	0
3AN3	*Pseudomonas*	γ- proteobacteria	0		0	0	0	0	0
3SC1	*Pseudomonas*	γ- proteobacteria	0		**1**	0	0	0	0
3SC2	*Pseudomonas*	γ- proteobacteria	0		0	0	**1**	0	0
3SC3	*Pseudomonas*	γ- proteobacteria	0		0	0	0	0	0
4ALev1	*Pseudomonas*	γ- proteobacteria	0		0	**1**	0	0	0
4SC1	*Pseudomonas*	γ- proteobacteria	0		**1**	0	0	0	0
4SC2	*Pseudomonas*	γ- proteobacteria	0		0	0	0	0	0
4SC3	*Pseudomonas*	γ- proteobacteria	0		0	0	0	0	0
4SL1	*Pseudomonas*	γ- proteobacteria	0		0	0	0	0	0
4SL2	*Pseudomonas*	γ- proteobacteria	0		0	0	0	0	0
4SLev1	*Pseudomonas*	γ- proteobacteria	0		**1**	**1**	0	**1**	0
4SLev2	*Pseudomonas*	γ- proteobacteria	0		0	**1**	0	0	0
4SN1	*Pseudomonas*	γ- proteobacteria	**1**	**1**	0	0	0	0	0
4SN2	*Burkholderia*	β- proteobacteria	0		0	**1**	0	0	0
5ALev1	*Pseudomonas*	γ- proteobacteria	0		0	**1**	0	0	0
5ALev2	*Janthinobacterium*	β- proteobacteria	0		0	**1**	0	0	0
5AN1	*Flavobacterium*	Flavobacteria	0		0	0	0	0	0
5AN2	*Pseudomonas*	γ- proteobacteria	0		0	**1**	0	0	0
5AN3	*Pseudomonas*	γ- proteobacteria	0		0	**1**	0	0	0
6AL1	*Pseudomonas*	γ- proteobacteria	0		0	**1**	0	0	0
6AL2	*Serratia*	γ- proteobacteria	0		**1**	0	0	0	0
6AL3	*Pseudomonas*	γ- proteobacteria	0		0	0	0	0	0
6AN1	*Serratia*	γ- proteobacteria	0		**1**	**1**	0	0	0
7Alev1	*Pseudomonas*	γ- proteobacteria	0		0	**1**	0	0	0
7ALev2	*Chryseomonas*	γ- proteobacteria	0		0	0	0	0	0
7AN1	*Serratia*	γ- proteobacteria	**1**	**1**	0	0	0	0	0
7AN2	*Pseudomonas*	γ- proteobacteria	0		0	**1**	0	**1**	0
7AN3	*Chryseomonas*	γ- proteobacteria	0		0	0	0	0	0
8ABCSA1	*Pseudomonas*	γ- proteobacteria	0		0	**1**	0	0	0
8Alev1	*Pseudomonas*	γ- proteobacteria	0		0	**1**	0	0	0
8ALev2	*Pseudomonas*	γ- proteobacteria	0		0	0	0	0	0
8ALev3	*Pseudomonas*	γ- proteobacteria	0		0	**1**	0	0	0
8AN1	*Pseudomonas*	γ- proteobacteria	0		0	0	0	0	0
8AN2	*Janthinobacterium*	β- proteobacteria	0		0	0	0	0	0
9Alev1	*Pseudomonas*	γ- proteobacteria	0		0	0	0	0	0
9Alev2	*Pseudomonas*	γ- proteobacteria	0		0	**1**	0	0	0
9AN1	*Pseudomonas*	γ- proteobacteria	0		**1**	0	0	0	0
9AN2	*Burkholderia*	β- proteobacteria	0		**1**	**1**	0	0	0
9AN3	*Pseudomonas*	γ- proteobacteria	0		0	**1**	0	0	0
9AN4	*Pseudomonas*	γ- proteobacteria	0		0	0	0	0	0
9AN5	*Pseudomonas*	γ- proteobacteria	0		0	0	0	0	0
9SC1	*Burkholderia*	β- proteobacteria	0		0	0	0	0	0
9SN1	*Pseudomonas*	γ- proteobacteria	**1**	**4**	0	0	0	0	0
9SN2	*Pseudomonas*	γ- proteobacteria	0		0	0	0	0	0
9AL34	*Aranicola*	γ- proteobacteria	**1**	**9**	0	0	0	**1**	0
10AL1	*Enterobacter*	γ- proteobacteria	**1**	**5**	0	0	0	0	0
10AL3	*Serratia*	γ- proteobacteria	0		0	0	0	0	0
10AL4	*Pseudomonas*	γ- proteobacteria	0		0	0	**1**	0	0
10AN2	*Pseudomonas*	γ- proteobacteria	0		0	0	0	0	0
10SL1	*Pseudomonas*	γ- proteobacteria	0		0	0	0	0	0
10SL2	*Pseudomonas*	γ- proteobacteria	**1**	**5**	0	0	**1**	**1**	0
10SL3	*Pseudomonas*	γ- proteobacteria	**1**	**2**	0	0	**1**	0	0
10SL4	*Pseudomonas*	γ- proteobacteria	**1**	**3**	0	0	0	**1**	0
10SN1	*Pseudomonas*	γ- proteobacteria	0		0	0	0	0	0
10SN2	*Pseudomonas*	γ- proteobacteria	0		0	0	**1**	0	0
10SN3	*Pseudomonas*	γ- proteobacteria	0		0	0	**1**	0	0
10SN4	*Pseudomonas*	γ- proteobacteria	0		0	0	0	0	0

The first number of each isolate corresponds to the sampling site (from 1 to 10, see Table1).

a Mutations respect to the clinical alleles (AN DQ247972, EU855788 and CP000650). The *intI1* gene of the isolates1AC2 and 1SL5 are 100% identical to AN CP000650 and DQ247972, respectively.

A previous study from Australia found a prevalence of *intI1* genes in non-clinical isolates of β*-*proteobacteria strains in a similar culture-based method [1]. They also found a great dispersion of *qac* genes in environmental samples, and proposed that selection for *qac* resistance before the antibiotic era contributed to the mobilization and widespread of class 1 integrons among the environmental Proteobacteria. They argued that when antibiotics began to be administrated it would be almost inevitable that class 1 integrons would come to play a major role in the dissemination of antibiotic resistance [50].

With the information available from ours and other studies [48], [50], it is possible to build a hypothesis on the role of environmental γ and β-proteobacteria as sources of clinical *intI1* genes. In addition, we found that at each sampling site positive for *intI1*, IS*CR1* was also detected and at least one *sul1* and/or *qacE1/qacE*Δ*1* gene was identified. Although the succession of molecular steps involved in the acquisition of components of the genetic platforms of class 1 integrons circulating in hospitals nowadays (Figure 1) could not be determined, our study showed a scheme in which the γ and β proteobacteria harbouring *intI1* genes share habitats with several other genera belonging to γ, β-proteobacteria, Actinobacteria and Bacteroidetes, which in turn have the IS*CR1*, *sul1*, *tniC* and/or *qacE1/qacE*Δ*1* genes. These genes could have been co-acquired in one bacterial cell by mechanisms associated with lateral genetic transference and later selected by antimicrobial pressure within clinical settings and/or by human activities.

### The *Sul1* Gene is the Only Genetic Marker Associated with Urbanization

**Figure 3:**
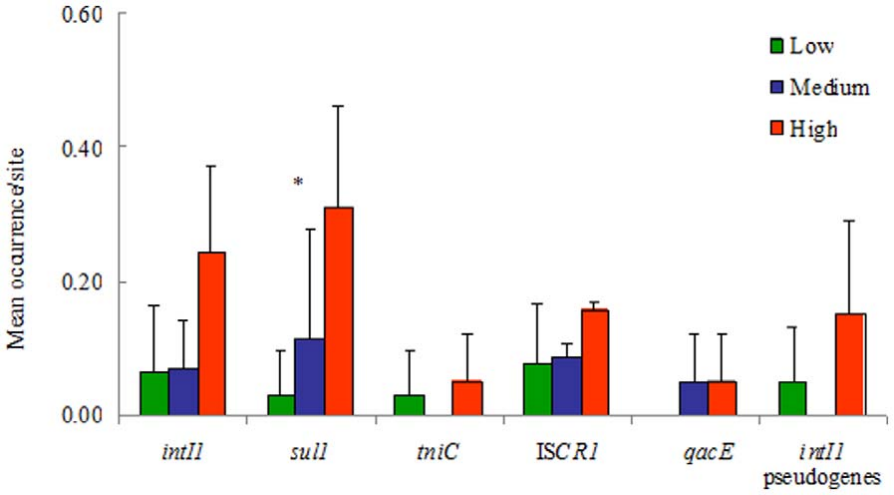
Mean occurrence (+SD) of LGE and ARG genes and pseudogenes in sites with different degrees of urbanization. Most genes showed trends towards high occurrences in highly urbanized areas, but only *sul1* showed statistical significance in this trend (r_s_ = 0.74, p = 0.01).

**Figure 4:**
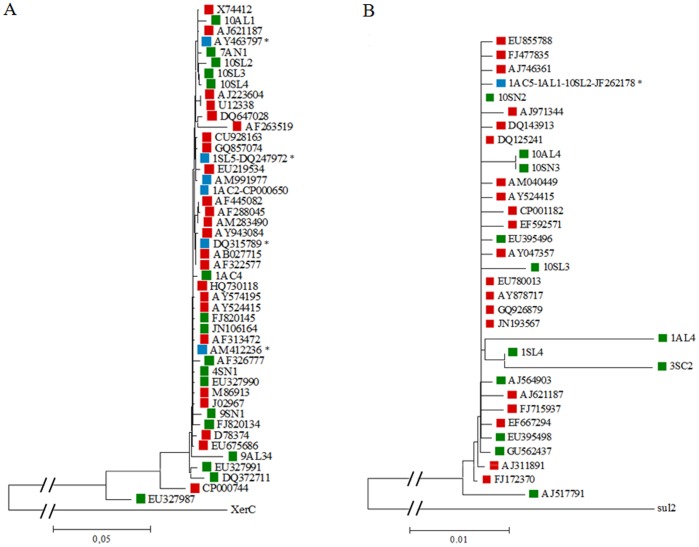
Phylogenetic trees for *intI1* (A) and *sul1* (B) genes. Sequences obtained in this study and alleles deposited in the GenBank were used. The phylogram was obtained with the CLUSTALW application in MEGA v 5.05 program with default parameters. Alleles were indicated with either the isolated name (sequences obtained in this work) or the accession number (GenBank sequences). The source of each allele is shown by a coloured square (red is for clinical isolates, green is for environment isolates and blue is for alleles that have been identified in both clinical an environmental isolates. Asterisks indicate the most common clinical alleles of *intI1* (A) and the allele of *sul1* embedded in the most common 3′CS of clinical integrons (B). The outgroup branch has been reduced in order to appreciate better the other branches.

The frequency of occurrence of *sul1* was significantly and positively related to the level of urbanization, whereas the other genes, *intI1*, IS*CR1*, *qacE1*/*qacE*Δ*1* and *tniC*, were not significantly related to this variable (p>0.05) (Figure 3; Table 3). All 10 *sul1* genes obtained by PCR were sequenced and 7 of them exhibited more than two mutations in 581-bp length compared to the *sul1* from clinical isolates (accession number JF262166). In addition, high sequence diversity of the *sul1* gene (83% identity compared to the *sul1* sequence in accession number JF262166) was found in one strain isolated from a site with low-level anthropogenic disturbances (3SC2 isolate, Table 2). In order to analyse the relationship between “environmental” and “clinical” types of genes we performed a phylogenetic analysis with *sul1* alleles from the Genbank and from our work (Figure 4). More than 30 alleles of the *sul1* gene were identified in this analysis. Only the *sul1* allele from the 3′-CS of the integrons was found in both clinical and non-clinical isolates.

**Table 3:** Wald statistic values obtained from the generalised lineal model analyses that were applied to the relationships between gene frequencies and 3 independent variables.

	*intI1*	*intI1* mutations	*intI1* pseudogen	*tniC*	IS*CR1*	*sul1*	*qacE*
Intercept	0.01	10.59	1.35	0.01	0.01	1.80	0.10
Substrate	2.09	1.94	0.12	0.79	0.76	0.05	0.10
Taxa	0.01	0.01	0.17	0.01	0.01	2.94	6.84*
Urbanization	0.01	0.01	0.70	0.01	0.01	7.55*	0.74

* represents p<0.05.

The high frequency of the *sul1* genes in urban sites could be the consequence of gene flow of the “clinical” *sul1* allele from the hospital towards the open environment to which are added the *sul1* alleles of non-clinical strains that could be in turn maintained by the presence of contaminants that co-select for sulphonamide resistance.

### A Wide Dissemination of the *qacE1*/*qacE*Δ*1* Gene was Found Between Taxa and Between Different Degrees of Urbanization in Patagonia

The distribution of the other ARG in this study, the *qacE1*/*qacE*Δ*1* gene, was wider among the environmental samples than found for *sul1*. Although we found that the *qacE1*/*qacE*Δ*1* gene was present in 28 out of 98 isolates and in 12 bacterial genera, this gene was not significantly related to “clean” or to urban sites (p>0.05) (Figure 3). We sequenced all *qacE1*/*qacE*Δ*1* amplicons, which exhibited 100% identity in 241 bp to the clinical allele (accession number HM999792). The high frequency of *qac* gene cassettes (*qacE*, *qacG* and *qacH*) found in non-clinical samples from Australia [51] allowed the authors to propose a relevant role for the *qacE1* gene cassette in the origin of the most common genetic platform of the clinical class 1 integrons. Following their hypothesis, the common ancestor of class 1 integrons was embedded in a Tn*402*-like transposon harbouring a complete *qacE1* gene cassette within the VR-1; this gene was subsequently deleted by insertion of the *sul1* gene and converted into the well-known *qacE*Δ*1* of the clinical 3′-CS of class 1 integrons [1], [52], [53]. Recently, an environmental permafrost strain which was presumed to date from to 15.000–40.000 years ago with a typical 3′-CS of clinical class 1 integrons was found in Siberia [54]. If this finding is not a contamination, it is likely that the 3′-CS has been in nature and probably maintained without interaction with human activities before the antibiotic era.

Clearly, no matter which genetic platform contains the *qacE1*/*qacE*Δ*1* gene, its dissemination as either a cassette or a pseudogene between clinical and natural communities is widespread around the world since a long time ago [1], [51], [53], [54] [this work], emphasizing the adaptive role that it possess for a large variety of genomes, habitats and possibly different types of stressors.

### Multiple Interactions Define the Ecological and Molecular Behaviour of Each ARG

While we found that the *sul1* and *qacE1*/*qacE*Δ*1* genes were usually located separately in our non-clinical isolates, both ARGs are embedded in the widespread 3′-CS of class 1 integrons when they are detected in clinical samples [1], [53]. We found both ARG together in only one isolate, 1AL4, which corresponds to *Streptomyces* spp. (phylum: Actinobacteria). The sequence revealed 100% identity over 822 bp with the array of the 3′-CS (accession number EU118148). This finding is probably the result of the flow of clinical strains harbouring class 1 integrons with the typical 3′-CS from the hospital to site 1. It is likely that a lateral genetic transfer event to the strain 1AL4 Actinobacteria has happened since this species is rarely isolated from human infections.

Previous reports have shown that sulphonamide and quaternary ammonium compound resistances are usually found in bacterial isolates from natural communities [40], [51], [55], [56], [57]. Also, very relevant for the evolution of multidrug isolates, both ARGs have been identified as possessing the potential to co-select for multidrug resistance in non-clinical and clinical samples [40], [56], [57], [58], [59].

However, both ARGs differ from functional, taxonomic distribution and molecular perspectives when they are analysed separately. It is well known that the *qacE1* gene is a mobile element since it has all of the features of a gene cassette, whereas the *sul1* is an open reading frame not associated with an *attC* site. Thus, it is likely that the mobility conferred by the system’s integron/cassette could be one reason for the widespread dissemination of *qacE1* within natural communities and genomes. The *qacE1*/*qacE*Δ*1* genes from our non-clinical samples were 100% identical to the 3′-CS of clinical class 1 integrons, showing a different molecular pattern to the *sul1* gene. For the latter gene it is possible to distinguish its “clinical” or “environmental” origin on the basis of the different nucleotide sequences, as also shown for alleles of *intI1*.

The results of this study showed that the *sul1* and *qacE1*/*qacE*Δ*1* genes have a different distribution between sites with different degrees of urbanization in Patagonia and diverse behaviour from a molecular perspective, suggesting that multiple interactions define the abundance of each type of ARG at a particular site. So these factors need to be analysed in individual studies for each antimicrobial resistance gene.

### Non-clinical Samples from Patagonia are a Reservoir of IS*CR1*

The IS*CR1* gene was found in γ and β-proteobacteria and in Actinobacteria isolates (n = 11) (Table 2), and it was common in *Pseudomonaceae* (8 out of 11 positive isolates), which is the first description of this site-specific recombinase gene in non-clinical samples. The sequence of 475 bp from the 11 IS*CR1*-positive isolates revealed 100% identity with the clinical allele (EU722351). In a previous study from our laboratory on clinical isolates, this gene was present in 40% of 130 *Enterobacteriaceae* strains and only in 1% of 100 *Pseudomonas aeruginosa* isolates (data not shown), suggesting a different taxonomic distribution between clinical isolates compared to natural communities. The high frequency exhibited by IS*CR1*, as well as its distribution in several taxa in Patagonian samples, could be evidence of the important role of the open environment as a reservoir of this gene in our geographical region. The IS*CR1* gene was found in bacterial cells in the same sampling sites where isolates with *intI1*, *qacE1/qacE*Δ*1* and/or *sul1* were also identified that ensures an encounter among cells and a putative transference of genes. This pool of genes could be the source for the emergence of the first strain harbouring *bla*_CTX-M-2_ associated with IS*CR1* on the genetic platform of a complex class 1 integron that had emerged in a clinical isolate in Argentina in 1989 [31], [60].

### Abundance and Flow of the *intI1* Gene in Environmental Samples

We found a frequency of 11.2% *intI1* genes in 98 isolates by plating on nutritive agar without antibiotics in 5 out of the 10 sampling sites. This frequency is relatively high in comparison to those obtained in previous studies. Rosewarne *et al*. [42] found only 0.5% of positive strains (4/790 isolates) and Stokes et al. [1] found 2.1% (4/192 isolates) in a similar model of a culture-based isolation of non-clinical strains without antibiotics in Australia. These contrasting results could be the consequence of different methodologies of isolation, types of habitats, bacterial communities, and also phylogenetic patterns could be involved in the abundance of this gene in different geographic regions. When we analysed the molecular features of the *intI1* genes from our study, we identified two “clinical” genes of the *intI1* gene that were found in the sampling site with the highest level of urbanization (site 1, Ushuaia city). These two “clinical” *intI1* genes could belong to different clinical strains since they were different alleles obtained from different species of bacteria: sample 1SL5 was from a *Pseudomonas* spp. (accession number DQ247972 with 22.13% of *intI1* genes from Genbank) and sample 1AC2 was from an *Aeromonas* spp. (CP000650 with 2.55% of *intI1* genes from Genbank). The remaining nine alleles, which all were “environmental” *intI1* genes (n = 5 in *Pseudomonas* spp., n = 1 in *Vibrio* spp. and n = 3 in *Enterobacteriaceae*) (Table 2), showed sequence diversity with novel mutations that have never been described before; neither in non-clinical nor in clinical *intI1* genes (Figure 4).

The *intI1* gene flow from the hospital to the open environment has been well established [47], [48], [50]. Based on the large number of *intI1* alleles found in non-clinical samples [1], [48][this study], in clinical isolates (from GenBank, up until August 2010, Figure 4), and also found in both types of environments (Figure 4), we propose that at least two different routes of the acquisition of class 1 integrons could have interacting in hospitals during the antibiotic era: on one hand, the most common alleles of *intI1* (accession numbers DQ247972, AY463797, AM412236 and DQ315789) must have been the first that were introduced in the hospital niche and, thereafter, were continuously selected by the pressure of antibiotics; and, on the other hand, in certain circumstances, “environmental” *intI1* genes must have been taken from natural communities and thereby began their propagation and circulation in the clinical habitat under antimicrobial pressure. In our understanding, this flow of genes from the open environment to hospitals is also evidenced by the new and unusual 3′ends of class 1 integrons that have been described in sporadic isolates worldwide. Examples of this are strains harbouring the IRt of Tn*402* [61] or IS*440*-*sul3*-*orf1*-IS*26* [26] instead of 3′-CS. In fact, this latest genetic platform has been described as harbouring the *qacH* gene cassette within the VR-1, which has been detected very frequently in non-clinical samples [26], [51]. The high frequency of different *intI1* alleles found in clinical and non-clinical isolates shown in the phylogenetic tree of the Figure 4 also suggests that *intI1* gene flow between human activities and the environment occurs in both directions.

### The High Frequency of *intI1* Pseudogenes Reveals a Similar Genomic and Ecological Behaviour for Integron Integrases

The *intI1* pseudogenes were found in 9 out of the 10 sampling sites of Tierra del Fuego Island. From a total of 30 *intI1*-positive isolates, the frequency of occurrence of *intI1* genes was 11.2% (11/98) if the entire *intI1* gene sequence is considered, and it was 19.4% (19/98) if only the amplification of the integron integrase motif [62] is taken into account (Table 2). While the complete sequence of the *intI1* gene was only detected in γ-proteobacteria, the *intI1* pseudogenes were also found in two β-proteobacteria isolates and in one Actinobacteria isolate (1SC3). Thus, the range of dispersion of *intI1* genes was increased between different taxa and between different sampling sites due to the identification of *intI1* pseudogenes. However, this genetic marker was not related to urbanization, which demonstrates that the entire *intI1* gene and its pseudogenes exhibit a similar ecological behaviour.

A previous bioinformatics study of all families of integron integrase genes found that 1/3 of *intI* were pseudogenes [63]. The prevalence of *intI1* pseudogenes in our non-clinical samples was two times higher than that of the entire *intI1* gene. This greater frequency of *intI1* pseudogenes observed in this study is evidence of the significant adverse effects produced by the entire gene in many different bacterial genomes. On the other hand, this widespread dissemination also highlights the fact that class 1 integrons possess a successful mechanism for spreading among natural bacterial communities. In addition, the *intI1* pseudogenes have a different pattern of distribution if we compare the natural with the clinical communities. The bioinformatics study we performed on DNA sequences from clinical strains in Genbank (up until August 2010) revealed that only *Corynebacterium diphtheriae* (accession number BX248353) has an *intI1* pseudogene. Thus, the low frequency of *intI1* pseudogenes and, therefore, the high frequency of entire *intI1* genes in the clinical isolates from GenBank revealed that the genomes circulating in clinical communities have possibly been selected because they have a genomic plasticity that facilitates maintenance of the entire *intI1* gene.

### The *intI1* Genes Isolated from the Open Environment were not Related to Urbanization

Although a weak trend towards a high occurrence of *intI1* genes in urban areas was observed, there were no statistical correlations between the mean occurrence per site of the *intI1* genes and its pseudogenes and the three degrees of urbanization (p>0.05) (Figure 3; Table 3). In other words, “environmental” *intI1* genes were not significantly more abundant in anthropic environments than in remote areas from urban centres.

However, previous reports showed that sites close to human activities have a higher frequency of *intI1* genes, as a result of the *intI1* flow from clinical samples to the open environment [42], [64]. The discharge of genes should be maintained by the release of antibiotics at urban sites or by the presence of contaminants as metals [37], [42], [64]. The process of co-selection, would be involved in the maintenance of *intI1* genes [42], [47], as it is the case for transposon Tn*21*, which possesses determinants of resistance to mercury and usually has a class 1 integron embedded in its genetic platform [65]. Several studies evidenced the flow of class 1 integrons from humans to wastewater treatment plants, rivers, soil and domestic and wild animals [64], [66], [67], [68], [69], [70], [71], [72]. From a molecular perspective, a similar scenario was found in our study, since the “clinical” alleles of the *intI1* gene were only found in sites with a high level of urbanization.

When class 1 integrons were searched for in *E. coli* strains isolated from several animal populations subjected to different degrees of anthropogenic disturbance, the abundance of *intI1* was found to correlate with the closeness to humans [64]. Skurnik *et al*. [64] explained the absence of class 1 integrons in wild animals as a result of never having been exposed to humans. However, the absence of class 1 integrons in *E. coli* strains not exposed to human disturbances can be expected since it has been suggested that the genome of *E. coli* is not able to acquire or maintain class 1 integrons without antibiotic pressure [55]. In other study, Rosewarne *et al.* (2010) [42] compared the abundance of *intI1* in catchments with different levels of human disturbance from the Greater Melbourne area of Victoria, Australia, and found a strong positive relationship between the frequency of occurrence of this gene and heavy metal pollution. One explanation for our findings is that the apparently “clean” sites of Tierra del Fuego Island in fact receive or have received some source of pollution (flow of *intI1*-positive clinical strains, antibiotics and/or heavy metals) that is not associated with the level of urbanization. Some sites are visited by tourists, but most only have a very low frequency of visitors in the months when there is no snow. Nor is it likely to be explained by current pollution levels from heavy metals, which are concentrated in areas close to the cities in this region [73], [74]. Moreover, the existence of pollution sources in the past is unlikely as the island of Tierra del Fuego has remained largely untapped for decades because of its geographical position and difficulty of access. Therefore, although cases of contamination could have been sporadic in the past, these could not explain the persistence of *intI1* in non-urban regions. Another interpretation for our results it is that the lack of correlation found in our study could be due to the small sample size; however, the frequency of occurrence of *intI1* in “clean” sites of Tierra del Fuego Island was sufficiently large to deserve attention. Another hypothesis for explaining the differences between studies regarding the incidence of *intI1* in “clean” sites could be that there are regions of the Earth in which some lateral genetic transfer mechanisms might be more abundant, due to historical processes of regional scale. Independent phylogenetic processes may have caused the *intI1* gene to be differentially adapted to different bacterial genomes. The consequence would be that, in certain regions, bacterial species maintain the *intI1* gene irrespective of anthropogenic pressure. These type of analysis, based on molecular and ecological studies but on a global scale, could be helpful for disentangling the multiple factors that are involved in the flow and maintenance of the *intI1* gene between areas of human activities and natural communities.

### Conclusions

Simultaneous analyses at ecological and molecular levels appeared to be a successful strategy for elucidating the role of each component of the genetic platforms associated to antimicrobial resistance of class 1 integrons.

We found that both ARGs (*sul1* and *qacE1/qacE*Δ*1* genes), which are usually embedded in the most common genetic platform of class 1 integrons within clinical habitats, showed different ecological and molecular behaviours in natural communities. While the presence of the *sul1* gene was the only component of the genetic platforms of class 1 integrons related to urbanization, the *qacE1/qacE*Δ*1* gene was found to be widespread in natural communities with different degrees of anthropogenic disturbances, which highlights the adaptive role of this gene to several different habitats. The LGE (*intI1*, IS*CR1* and *tniC* genes) can exhibit high levels of diversity and different levels of persistence depending on the habitat and regions of the world. Ecological analysis showed that the *intI1* gene, as well as the IS*CR1* genes, which are relevant mechanisms involved in the spreading of multidrug resistance mechanisms in clinical isolates in Argentina and worldwide, were not associated with urbanization in the Patagonian samples. A total of 30/98 *intI1*-positive isolates were identified, with a high frequency of *intI1* pseudogenes (19/98), which suggests that although *intI1* has a deleterious impact within several genomes, it can easily be disseminated throughout natural bacterial communities. We cannot rule out the possibility that the high percentage of *intI1* and IS*CR1* genes that we found in the natural communities may be one of the factors that contributes to the increasing frequency of antimicrobial resistance isolates that have characterized Argentina for decades.

The main conclusion of this study is that the ability of natural bacterial communities to act as a reservoir and source of multidrug resistance mechanisms cannot be described by a general process but depend on multiple factors operating at molecular, ecological, phylogenetic and historical levels.

## Materials and Methods

### Study Area

The study was conducted in the south-eastern portion of Tierra del Fuego Island (Figure 2). The area lies within the Sub-Antarctic Deciduous Beech Forest, which is characterized by two species of southern beech, *Nothofagus pumilio* (Lenga) and *Nothofagus betuloides* (Guindo) [75]. Its climate belongs to the sub-polar oceanic type. Temperatures are cold all year round, with an average annual temperature of 5.7°C and low annual temperature variations, ranging from −0.3°C in July to 9.4°C in January. There are two urban sites: Ushuaia city with 80,000 inhabitants on the southern coast of the island, bathed in the Beagle Channel, and Tolhuin, a town of about 8000 inhabitants. The island was only colonized at the end of the nineteenth century.

All necessary permits for the described field studies were obtained from Clotilde Lizarralde (Director of the Planning Department in the science and technology area of the province) and Laura Malmierca (Tierra del Fuego National Park Management).

### Definition of the Degree of Urbanization

Samples were collected in 10 sites that were selected according to three distinct levels of anthropogenic disturbance (Figure 2 and Table 1). The degree of urbanization was quantitatively estimated by counting the number of buildings and roads that were contained in a 1 km circular area around each site. These estimations were conducted using satellite images provided by Google Earth. We defined a high level of urbanization as being when the number of buildings was greater than 50 and the number of routes greater than 5. We defined a low level of urbanization as being when the number of buildings was less than 5 and the number of routes less than 2. Two sites were located in Ushuaia City, in the mouths of the Pipo (Site 1) and Ushuaia (Site 10) rivers; three sites were in Tierra del Fuego National Park, at a stream that ends in Ensenada Bay (Site 7), at an upper portion of Pipo River (Site 8) and at Ovando River (Site 6); two sites were at Escondido Lake, one on the shore of the lake where a hotel is located (Site 4) and the other one at a stream that finishes in this lake (Site 5); two sites were along the Provincial Road N° 26 in a mountainous area south-east of Fagnano Lake, one of which was in the intersection of this road with Turbio River (Site 2) while the other was at Turbera Maucasen (Site 3); and site 9 was located in Moat Farm, in a stream close to the sea (Figure 2). Sites 3 to 9 had no history of clinical or industrial activities. Animal husbandry in the studied areas is minimal, and there is not systematic records of the use of antibiotics on domestic animals. The sites of Turbio River and Moat Farm are occasionally visited, mainly in the summer. Sites 1 and 10 were categorized as being highly disturbed, sites 3 and 4 were categorized as being medium-level disturbed, and the others were categorized as being low-level disturbed sites (Table 1).

### Sampling Techniques

Samples were collected at each site between 20^th^ January and 7^th^ February 2006. Shallow freshwater sediment and soil samples from the shore were plated on nutritive agar medium (Britania, Argentina) without a selection of antibiotics. The plates were incubated at 4°C for 8 days, after which all individual colonies from each site and from each plate were plated again in nutritive agar and incubated at 4°C for 4 days. Then, each colony was picked out and place onto Luria Bertani broth and incubated at 4°C for 48 h.

### Molecular Analysis

Because the goal of this work was to analyse the genetic platforms of class 1 integrons, we worked with a culture-dependent technique in order to identify how many ARG or LGE could be harboured in a single strain. The isolates were identified using standard biochemical tests, microbiological test strips (API20NE-Biomerieux, France) and sequencing of 16 S RNA using universal primers [76]: *Arthrobacter* spp. (n = 6), *Aeromonas* spp. (n = 4), *Vibrio* spp. (n = 2), *Enterobacter* spp. (n = 2), *Streptomyces* spp. (n = 1), *Microbacterium* spp. (n = 1), *Pseudomonas* spp. (n = 56), *Micrococcus* spp. (n = 1), *Janthinobacterium* spp. (n = 7), *Yersinia* spp. (n = 2), *Flavobacterium* spp. (n = 3), *Paenibacillus* spp. (n = 1), *Serratia* spp. (n = 5), *Burkholderia* spp. (n = 4), *Chryseomonas* spp. (n = 2), *Aranicola* spp. (n = 1).

Then, total DNA was extracted and Polymerase Chain Reaction (PCR) amplifications were carried out in 50 µl volumes containing 10 ng of DNA, 1× PCR buffer (Promega, USA), 0.2 mM of dNTPs mix (Genbiotech, Argentina), 0.4 µM of each primer (Genbiotech, Argentina) and sterile distilled water, and *Taq* DNA polymerase (Promega, USA) was added (0.25 U). For the detection of the *intI1* gene, two strategies were used by amplifying a PCR fragment of 925-bp length (5′-cgaggcatagactgtac-3′ and 5′-ttcgaatgtcgtaaccgc-3′) [32] and another of 483-bp length (5′-acatgcgtgtaa atcatcgtcg-3′ and 5′-gggtcaaggatctggatttcg-3′) [77] that included the additional motif that it is conserved among integron integrases [62], [78], [79]. When only the 483-bp amplicon was obtained, the HS915 primer (5′-cgtgccgtgatcgaaatccag-3′) in conjunction with the HS916 primer (5′-ttcgtgccttcatccgtttcc-3′) [80] was used in order to detect a putative, whole *intI1* gene. The detection of *intI1* and *intI1* pseudogenes was performed by two people at different times with independent DNA extractions and repeated at least twice. Also, the presence of *sul1* (5′-tttgaaggttcgacagc-3′ and 5′-gacggtgttcggcattct-3′) [81], *qacE1/qacE*Δ*1* (5′-gcgaagtaatcgcaacatcc-3′ and 5′- agccccatacctacaaagcc-3′) [30], IS*CR1* (5′-atggtttcatgcgggtt-3′ and 5′-ctgagggtgtgagcgag-3′) [32] and *tniC* (5′-ccgagggagagcagctt-3′′ and 5′-ccggtcacggtgcggcg-3′) genes were investigated in all strains. The PCR products were sequenced after purification using the Wizard SV Gel and PCR clean-up System kit according to the manufacturer’s directions (Promega, USA); sequencing was performed on both DNA strands using ABIPrism 3100 BioAnalyzer equipment (Applied Biosystems, USA). The nucleotide sequences were analysed using Genetics Computer Group (GCG) and Blast V2.0 software (http://www.ncbi.nlm.nih.gov/BLAST/).

### Definition of “Clinical” and “Environmental” *intI1* Alleles

We called non-clinical *intI1* genes those that were harboured by the strains isolated from water, sediment or soil in this work. The non-clinical *intI1* gene can also be an “environmental” or a “clinical” allele as defined by Gillings *et al.,* suggesting a putative source from natural or clinical communities, respectively [48].

### Bioinformatics Study of the *intI1* Gene

We identified 47 alleles from clinical samples in Genbank (up until August 2010); the most common alleles used for defining a “clinical” allele were those with the accession numbers DQ247972 (22.13%), AY463797 (19.15%), AM412236 (18.72%) and DQ315789 (17.02%).

### Phylogenetic Analysis

Phylogenetic evolutionary analysis for *intI1* and *sul1* sequences were conducted using MEGA v 5.05 software [82]. Sequences obtained in this study as well as alleles deposited in GenBank were included. These sequences were aligned using ClustalW application in MEGA with default parameters. The evolutionary history was inferred using the Neighbor-Joining method. The evolutionary distances were computed using the Maximum Composite Likelihood method. All positions containing gaps were eliminated.

### Statistical Analysis

The relationship between the environmental characteristics and the distribution of molecular components of antibiotic resistance were analysed using two statistical approaches (STATISTICA package). Generalized linear models (GLM) were applied with the following characteristics: (1) dependent variable: presence/absence of *intI1*, *sul1*, *tniC* and IS*CR1*, and number of *intI1* mutations; (2) independent variables: two categorical variables, substrate (water or soil) and taxa (γ-proteobacteria or other taxa) and one ordinal variable, degree of urbanization (1, 2 or 3); (3) assumed distribution of the dependent variable: binomial for presence/absence data and ordinal multinomial for mutations; (4) link function: logit.

The GLM was complemented with classic non-parametric tests. For the role of substrate and taxa in the presence of resistance genes, we used 2×2 contingency tables and Fisher’s exact tests, applied to frequencies of occurrence. For the effect of degree of urbanization, we applied Spearman’s rank correlations to mean occurrences of genes per site as a dependent variable.

### Nucleotide Sequence Accession Numbers

*IntI1* and *sul1* sequences were deposited at GenBank as accession numbers JN870902 to JN870912 and JX048595 to JX048604 respectively.
